# Efficacy and safety results from a randomized double-blind study comparing proposed biosimilar ABP 798 with rituximab reference product in subjects with moderate-to-severe rheumatoid arthritis

**DOI:** 10.1007/s10067-020-05305-y

**Published:** 2020-09-02

**Authors:** Gerd Burmester, Edit Drescher, Pawel Hrycaj, David Chien, Zhiying Pan, Stanley Cohen

**Affiliations:** 1 Department of Rheumatology and Clinical Immunology, Free University and Humboldt University Berlin, Charité—University Medicine Berlin, Charitéplatz 1, 10117 Berlin, Germany; 2 Veszprém Csolnoky Ferenc County Hospital, Veszprém, Hungary; 3 Rheumatology, Koscian Municipal Hospital, Koscian, Poland; 4 Amgen Inc., Thousand Oaks, CA USA; 5 Metroplex Clinical Research Center, Dallas, TX USA

**Keywords:** ABP 798, Biosimilar, Efficacy, Rituximab, Safety

## Abstract

**Background/objectives:**

ABP 798 is a proposed biosimilar to the originator biologic rituximab, an anti-CD20 monoclonal antibody. This comparative clinical study evaluated the pharmacokinetics (PK), safety, and efficacy of ABP 798 versus rituximab reference product (RP) in patients with moderate-to-severe rheumatoid arthritis (RA).

**Methods:**

Adults with moderate-to-severe RA with an inadequate response or intolerance to other disease-modifying anti-rheumatic drugs including 1 or more tumor necrosis factor inhibitor therapies (*n* = 311) received ABP 798, US-sourced rituximab RP (rituximab US), or EU-sourced rituximab RP (rituximab EU) (1000 mg, 2 weeks apart). At week 24, ABP 798- or rituximab EU-treated subjects received a second dose of the same treatment, while rituximab US-treated subjects transitioned to receive ABP 798. The key efficacy endpoint was DAS28-CRP change from baseline at week 24. Other efficacy endpoints included DAS28-CRP at other time points; ACR20, ACR50, and ACR70 criteria; and hybrid ACR. The rituximab RP groups were pooled for all efficacy endpoints since PK equivalence had been established between rituximab US and rituximab EU.

**Results:**

Clinical equivalence between ABP 798 and rituximab RP was established as the 90% confidence interval for DAS28-CRP change from baseline at week 24 fell within the prespecified equivalence margin (− 0.6, 0.6). Safety and immunogenicity profiles of ABP 798 were comparable across treatment groups and not affected by single transition from RP to ABP 798.

**Conclusions:**

Clinical equivalence in terms of efficacy, safety, and immunogenicity was established between ABP 798 and rituximab RP in this comparative clinical trial in patients with moderate-to-severe RA.

**Table:** 

**Key Points** *• ABP 798 provided similar efficacy as rituximab reference product (RP) in patients with moderate-severe rheumatoid arthritis.* *• The safety and immunogenicity profiles for ABP 798 were similar to those for the rituximab RP.* *• The single transition from rituximab RP to ABP 798 did not show differences in efficacy, safety, or immunogenicity.*

## Introduction

ABP 798^1^ is a proposed biosimilar to rituximab (Rituxan®, MabThera®) reference product (RP), a chimeric monoclonal immunoglobulin (Ig) G1 kappa antibody targeting the CD20 antigen expressed on B-cells [1, 2]. Rituximab exerts its effects primarily through induction of B-cell lysis upon binding to CD20; additional effects are mediated through complement-dependent cytotoxicity (CDC) and antibody-dependent cell-mediated cytotoxicity (ADCC) to a lesser extent [3]. Given that B-cells are critical for the pathogenesis of rheumatoid arthritis (RA), rituximab mediates its therapeutic effect in RA by targeted depletion of circulating and tissue B-cells [4].

Rituximab RP is approved for several indications, including non-Hodgkin lymphoma, chronic lymphocytic leukemia, moderate-to-severe RA, granulomatosis with polyangiitis and microscopic polyangiitis, and moderate-to-severe pemphigus vulgaris [1]. In moderately to severely active RA, rituximab is indicated in combination with methotrexate for the treatment of adult patients who have had an inadequate response to 1 or more tumor necrosis factor (TNF) antagonist therapies [1, 2].

Biosimilars are biologics that are highly similar to an already licensed biologic (originator biologic or RP) in terms of structure, purity, pharmacokinetics (PK), pharmacodynamics (PD), mechanism of action, potency, safety, and immunogenicity, and that have no clinically meaningful differences when compared with the originator or RP [5–10]. Demonstration of similarity requires a comparative stepwise approach of analytical (structural and functional) characterization and nonclinical and PK/PD studies of the proposed biosimilar and the originator or RP, and finally a comparative clinical confirmation of efficacy, safety, and immunogenicity in a representative indication using a sensitive population and sensitive endpoints [5–10].

ABP 798 has been shown to be structurally similar to rituximab RP [11]. ABP 798 has also been shown to be functionally similar to rituximab RP in terms of ligand binding, ADCC, and CDC [11]. As the next step in biosimilar development, a comparative clinical study was conducted to evaluate the similarity of ABP 798 to rituximab RP with regard to PK, PD, efficacy, safety, and immunogenicity in patients with moderate-to-severe RA. The PK equivalence and PD similarity between ABP 798 and rituximab RP (US-sourced rituximab [rituximab US] or EU-sourced rituximab [rituximab EU]) were established in patients with moderate-to-severe RA; these results have been reported separately [12, 13].

Here, we report the results of the clinical efficacy, safety, and immunogenicity similarity assessments of ABP 798 compared with rituximab RP in RA. In this study, the rituximab RP was acquired from both the US and the EU to satisfy the regulatory requirement of completing a scientific bridge between RP sourced from 2 regions during the demonstration of PK/PD equivalence. Per regulatory guidelines, RP is defined as that approved product in the local jurisdiction (i.e., US or EU based on the region of application), and the proposed biosimilar must be compared against each of the 2 locally sourced RPs [5, 10]. However, a single RP source may be used in confirmatory clinical studies if a scientific bridge has been established between the 2 locally sourced RPs in analytical and clinical PK/PD assessments.

## Materials and methods

### Subjects

Eligible subjects included men or women ≥ 18 and ≤ 80 years old with an RA diagnosis (based on meeting the 2010 American College of Rheumatology [ACR]/European League Against Rheumatism [EULAR] classification criteria for RA) [14] for a duration of at least 6 months. Other eligibility criteria included the presence of active RA (defined as ≥ 6 swollen joints and ≥ 6 tender joints based on 66/68 joint count excluding distal interphalangeal joints) at screening and baseline and erythrocyte sedimentation rate (ESR) ≥ 28 mm/h and/or serum C-reactive protein (CRP) > 1.0 mg/dL at screening. Subjects must also have had inadequate response [15] or intolerance to other disease-modifying anti-rheumatic drugs (DMARDs), including intolerance or inadequate response to 1 or more TNF inhibitor therapies. Subjects must have received methotrexate for ≥ 12 consecutive weeks and been on a stable dose (7.5 to 25 mg/week; oral or subcutaneous) for ≥ 8 weeks prior to receiving the investigational product (IP). Subjects taking oral corticosteroids (≤ 10 mg prednisone or equivalent per day) must have been on a stable dose for ≥ 4 weeks before IP initiation, and those receiving nonsteroidal anti-inflammatory drugs (NSAIDs) or low potency analgesics were to have been on a stable dose for ≥ 2 weeks prior to screening.

Exclusion criteria included a diagnosis of Class IV RA (according to ACR revised response criteria) [16], Felty’s syndrome (RA, splenomegaly, and granulocytopenia), history of prosthetic or native joint infection, active infection for which systemic anti-infectives were used ≤ 4 weeks or serious infection ≤ 8 weeks prior to first dose of IP, or malignancy ≤ 5 years (with the exception of treated and considered cured cutaneous squamous or basal cell carcinoma, in situ cervical cancer, or in situ breast ductal carcinoma).

### Study design

**Fig. 1:**
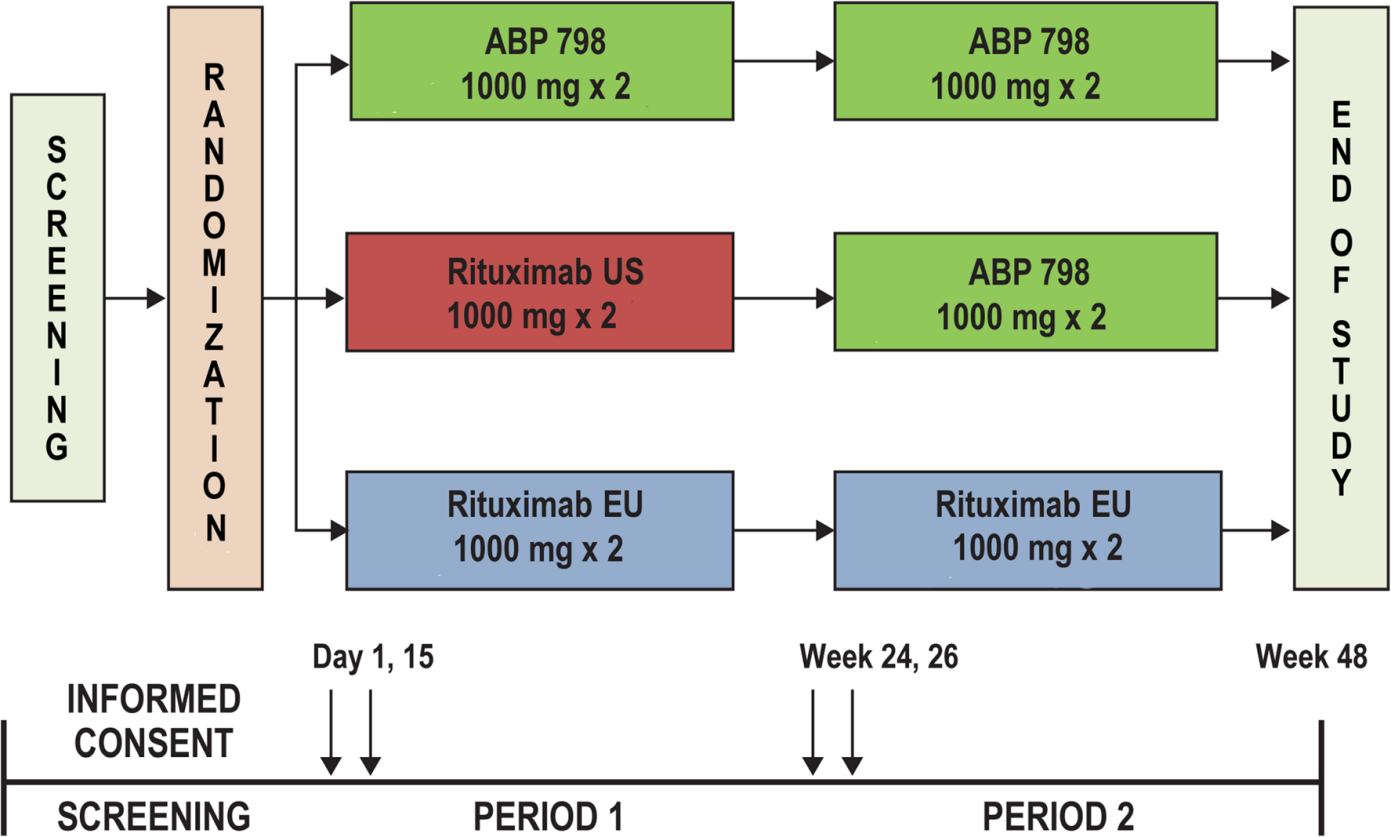
**a** Study design. **b** Subject disposition. Treatment was completed with the second dose, consisting of infusion at week 24 and at week 26. Study completion included completing treatment and final study assessments at end of study/week 48

This study was a randomized, double-blind, active-controlled study conducted at 57 centers in 6 countries (Bulgaria, Estonia, Germany, Hungary, Poland, and the USA; ClinicalTrials.gov NCT02792699) (Fig. 1a**)**. The study was conducted in accordance with the terms of the Declaration of Helsinki, Good Clinical Practice guidelines, and all applicable regulatory requirements.

Over the course of the study, 2 doses of IP were administered, consisting of 2 intravenous (IV) infusions of IP (1000 mg, administered 2 weeks apart). Subjects were randomized (1:1:1) to receive the first dose of either ABP 798, rituximab US, or rituximab EU in a double-blinded manner. At week 24, subjects in the ABP 798 or rituximab EU groups received the second dose of the same treatment, while those in the rituximab US group transitioned to receive ABP 798 for their second dose. Randomization was stratified by geographic region, seropositivity (rheumatoid factor [RF] positive and/or cyclic citrullinated peptide [CCP] positive vs. RF negative, and CCP negative), and number of prior biologic therapies used for RA (1 vs. > 1). Premedications were given according to local guidance and the approved product label; these included acetaminophen, an antihistamine, and methylprednisone 100 mg IV or equivalent 30 min before each infusion.

The total study duration was up to 52 weeks, including a screening period of up to 4 weeks. The first dose was administered on days 1 (week 0) and 15 (week 2), and the second dose on week 24 and week 26. The second dose may have been administered prior to week 24 in individual subjects (i.e., any time between week 16 and week 24), as deemed necessary by the investigator. The end of study (EOS) assessment was conducted at week 48 (or 24 weeks after the first infusion of the second dose for subjects re-treated before week 24).

### Study assessments

Disease assessments were conducted at baseline (day 1), week 8, week 12, week 24, week 40, and week 48 (EOS).

Subjects were monitored throughout the study for adverse events (AEs) and concomitant medication use. Clinical laboratory and vital signs were assessed at multiple time points during the study; these data are reported separately [13].

Blood samples for antidrug antibody (ADA) assessments were collected at baseline, week 2, week 24, week 30, and EOS.

### Efficacy endpoints

Efficacy assessments were secondary objectives in this study. The primary efficacy endpoint was the change from baseline of Disease Activity Score 28-joint C-reactive protein (DAS28-CRP) at week 24. Secondary efficacy endpoints included DAS28-CRP change from baseline at other time points; ACR20 (20% improvement in American College of Rheumatology core set measurements), ACR50 (at least 50% improvement compared with baseline), and ACR70 (at least 70% improvement compared to baseline) at weeks 8, 12, 24, 40, and 48 and hybrid ACR at weeks 8, 12, 24, 40, and 48.

### Safety endpoints

Safety endpoints included treatment-emergent adverse events (TEAEs) and serious adverse events (SAEs).

### Immunogenicity

The number and percentage of subjects who developed binding ADAs and neutralizing ADAs were assessed.

### Statistical analysis

Clinical equivalence was evaluated for the primary efficacy endpoint of change in DAS28-CRP from baseline at week 24 using the full analysis set (FAS), which included all subjects randomized in the study. A sample size of approximately 300 patients was to be randomized in this study; this provided > 90% power at a 2-sided 0.05 significance level to demonstrate equivalence in DAS28-CRP change from baseline at week 24 between the ABP 798 group and the pooled rituximab RP (rituximab EU + rituximab US) group with a margin ranging from − 0.6 to 0.6, assuming a standard deviation (SD) of 1.4 and a true mean difference of zero. The margin was chosen based on previously published findings [17, 18]. The approach for primary efficacy analysis of clinical equivalence depended on the results of the primary PK similarity assessment previously performed and reported [12, 13]. Because PK similarity had been established between rituximab US and rituximab EU, data from the 2 rituximab RP treatment groups (rituximab EU and rituximab US) were combined into a single-pooled rituximab RP group.

Clinical equivalence was tested by comparing the 2-sided 90% CI of the change from baseline at week 24 of DAS28-CRP between ABP 798 and the pooled rituximab RP groups with an equivalence margin of (− 0.6, 0.6). The 2-sided 90% CI was obtained using a repeated measures analysis in which data from all assessed post-baseline time points prior to week 24 were included.

Sensitivity analysis of the primary efficacy endpoint of DAS28-CRP change from baseline at week 24 was conducted on the per protocol analysis set (which included all subjects randomized in the study who had 2 full infusions of the first dose, completed the week 24 disease assessment, and did not experience a protocol deviation that affected their evaluation for the secondary objective of the study to assess clinical efficacy) to test the robustness of the primary efficacy analysis results. Other sensitivity analyses conducted included using an ANCOVA model for the week 24 DAS28-CRP change from baseline data adjusting for stratification factors and baseline DAS28-CRP results, analysis exploring the impact of additional baseline covariates, and a tipping point analysis to assess the sensitivity of results to the different assumptions of missing week 24 DAS28-CRP change from baseline data. Subgroup analyses of the primary efficacy endpoint were also conducted using the same repeated measures analysis model.

Analysis of the secondary efficacy endpoints of DAS28-CRP at other time points (weeks 8, 12, 40, and 48); ACR 20, 50, and 70 at weeks 8, 12, 24, 40, and 48; and hybrid ACR at weeks 8, 12, 24, 40, and 48 were summarized descriptively by treatment (ABP 798/ABP 798, rituximab EU/rituximab EU, or rituximab US/ABP 798).

Subject incidences of AEs, grade ≥ 3 AEs, fatal AEs, SAEs, adverse events of interest (AEOIs), AEs leading to discontinuation from IP or discontinuation from study, and subject incidence of ADAs were summarized using descriptive statistics. AEOIs are defined as clinically noteworthy events for a particular product or class of products that a sponsor may wish to monitor carefully. In this study, prespecified AEOIs included infusion reactions including hypersensitivity, cardiac disorders, serious infections, progressive multifocal leukoencephalopathy, hematological reactions, hepatitis B reactivation, opportunistic infections, hypogammaglobulinemia, severe mucocutaneous reactions, and gastrointestinal perforation. Safety laboratory parameters and vital sign measurements were summarized using descriptive statistics at each scheduled visit.

## Results

### Subject disposition

A total of 311 subjects were randomized and treated with IP (ABP 798, *N* = 104; rituximab EU, *N* = 104; rituximab US, *N* = 103); of these, 289 subjects completed treatment (infusions for the first dose [weeks 0 and 2] and the second dose [weeks 24 and 26]) (ABP 798/ABP 798, *n* = 97; rituximab EU/rituximab EU, *n* = 99; rituximab US/ABP 798, *n* = 93), and 282 competed the completed the study (week 48/EOS) (Fig. 1b). Fifty-five subjects (17.7%) received the first infusion of their second dose between weeks 16 and 24 (ABP 798/ABP 798, *n* = 21; rituximab EU/rituximab EU, *n* = 22; rituximab US/ABP 798, *n* = 12). Twenty-two subjects (7.1%) discontinued IP early (ABP 798/ABP 798, *n* = 7; rituximab EU/ rituximab EU, *n* = 5; rituximab US/ABP 798, *n* = 10). The most common reason for discontinuing IP was an AE (ABP 798/ABP 798, *n* = 3; rituximab EU/ rituximab EU, *n* = 1; rituximab US/ABP 798, *n* = 6). No major imbalance was observed among treatment groups.

#### Baseline characteristics and demographics

Key demographics and baseline characteristics were well balanced among the 3 treatment groups (Table 1**)**. The overall study population was 84.9% female, and 92.3% were white. The mean age was 55.9 years (SD, 10.91). In terms of disease characteristics, 246 (79.1%) subjects had a duration of RA of ≥ 5 years, with a mean duration of RA of 11.84 years (SD, 8.194; range, 0.6 to 44.0 years). The mean DAS28-CRP at study entry was 5.99 (SD, 1.015). The overall mean baseline weekly methotrexate dose was 16.4 mg (SD, 5.04), with a minimum dose of 7.5 mg and a maximum dose of 25 mg weekly. Approximately half of the patients (51.8%) were using glucocorticoids. In the total population, 186 (59.8%) subjects had 1 prior biologic therapy for RA, and 125 (40.2%) had > 1 prior biologic therapy for RA.

**Table 1:** Demographics and baseline characterizations

	ABP 798 (*N* = 104)	Rituximab EU (*N* = 104)	Rituximab US (*N* = 103)
Age (years), mean (SD)	54.6 (10.70)	56.8 (11.34)	56.4 (10.66)
Race, *n* (%)			
American Indian or Alaska Native	2 (1.9)	0 (0.0)	0 (0.0)
Asian (other)	0 (0.0)	2 (1.9)	1 (1.0)
Black or African American	5 (4.8)	3 (2.9)	10 (9.7)
White	97 (93.3)	99 (95.2)	91 (88.3)
Other	0 (0.0)	0 (0.0)	1 (1.0)
Sex, *n* (%)			
Female	90 (86.5)	91 (87.5)	83 (80.6)
Body mass index (kg/m^2^), mean (SD)	29.3 (6.4)	28.5 (7.1)	28.4 (6.3)
Prior biologic use for RA, *n* (%)			
0	0 (0.0)	0 (0.0)	1 (1.0)
1	54 (51.9)	58 (55.8)	55 (53.4)
> 1	50 (48.1)	46 (44.2)	47 (45.6)
Duration of RA (years)			
Mean (SD)	11.37 (7.400)	11.69 (7.945)	12.48 (9.186)
Median (range)	10.45 (0.6, 33.0)	9.0 (0.9, 39.0)	10.0 (0.7, 44.0)
Seropositivity, *n* (%)			
RF positive and/or CCP positive	85 (81.7)	91 (87.5)	88 (85.4)
RF negative and CCP negative	19 (18.3)	13 (12.5)	15 (14.6)
DAS28-CRP			
Mean (SD)	6.09 (1.035)	5.84 (1.006)	6.03 (0.997)
Median (range)	6.14 (3.1, 8.0)	5.92 (3.0, 8.0)	6.09 (2.7, 8.2)
Baseline MTX dose^a^ (mg/week),			
Mean (SD)	15.8 (5.29)	16.6 (5.11)	16.8 (4.68)
Median (range)	15.0 (8, 25)	15.0 (8, 25)	15.0 (8, 25)
Oral glucocorticoid use, *n* (%)			
Yes	58 (55.8)	52 (50.0)	51 (49.5)
No	46 (44.2)	52 (50.0)	52 (50.5)
Geographic region, *n* (%)			
Eastern Europe	59 (56.7)	58 (55.8)	59 (57.3)
North Europe	38 (36.5)	40 (38.5)	39 (37.9)
Western Europe	7 (6.7)	6 (5.8)	5 (4.9)

*DAS28-CRP* Disease Activity Score 28 joints-C-reactive protein, *MTX* methotrexate, *RA* rheumatoid arthritis, *SD* standard deviation

^a^Methotrexate 7.5-mg doses were received by 16 patients (USA, *n* = 5; Germany, *n* = 5; Hungary, *n* = 1; Poland, *n* = 1; and Bulgaria *n* = 4) who were randomized to ABP 798/ABP 798 (*n* = 8), rituximab EU/rituximab EU (n = 6), and rituximab US/ABP 798 (n = 2)

### Clinical efficacy

**Fig. 2:**
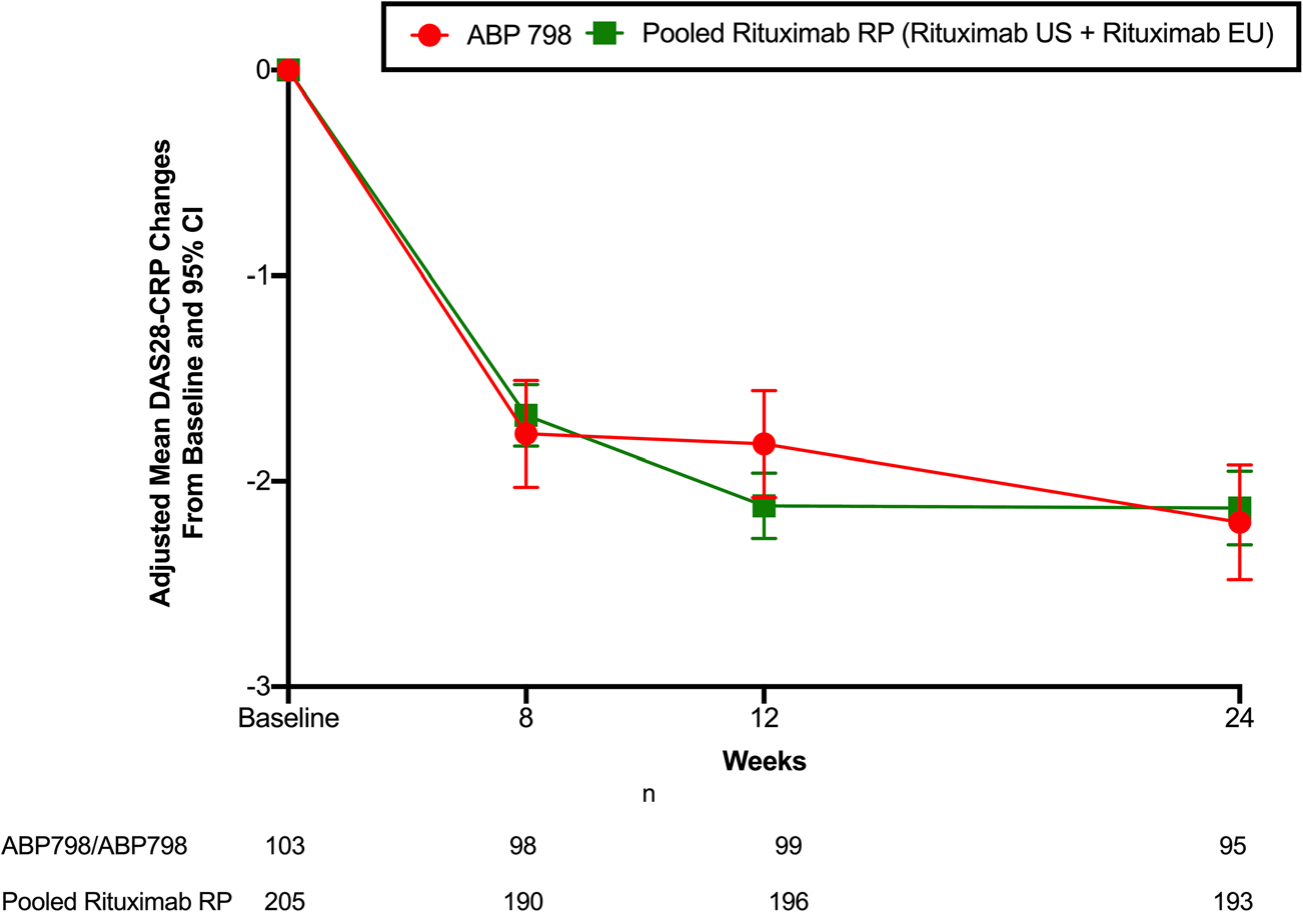
DAS28-CRP change from baseline at week 24 (primary endpoint)

PK analysis previously established the PK similarity between rituximab EU and rituximab US [12, 13]. Therefore, as per protocol specification, the rituximab US and rituximab EU groups were combined into a single reference group (pooled rituximab RP) for the primary assessment of clinical equivalence between ABP 798 and rituximab RP. The results of repeated measure analysis of DAS28-CRP change from baseline at week 24 based on FAS are presented in Table 2 and Fig. 2. The mean decrease from baseline in DAS-CRP was − 2.197 (SD, 1.3689) for ABP 798 and − 2.125 (SD, 1.3250) for pooled rituximab RP groups. The 90% CI (− 0.225, 0.264) for the mean difference at week 24 between ABP 798 and the pooled rituximab group was within the predefined equivalence margin (− 0.6, 0.6), thus allowing a conclusion of clinical equivalence between ABP 798 and rituximab. For the comparison between ABP 798 and rituximab US for change from baseline in DAS-CRP, the difference between the means was − 0.070 (90% CI, − 0.353, 0.213), and the difference between the means for ABP 798 vs. rituximab EU was 0.110 (95% CI, − 0.171, 0.392).

**Table 2:** Key efficacy endpoint of change from baseline in DAS28-CRP at week 24

	ABP 798 (*N* = 104)	Pooled Rituximab RP (EU + US) (*N* = 207)	Rituximab US (*N* = 103)	Rituximab EU (*N* = 104)
Mean (SD)	− 2.197 (1.3689)	− 2.125 (1.3250)	− 2.081 (1.3054)	− 2.168 (1.3491)
Difference between means (%)		0.020	− 0.070	0.110
90% CI (%)		− 0.225, 0.264	− 0.353, 0.213	− 0.171, 0.392

*CI* confidence interval, *EU* European Union, *N* number of subjects, *RP* reference product, *SD* standard deviation; *US* United States

Difference between means (ABP 798 − rituximab) and 90% CI for difference between means were based on repeated measure analysis with the DAS28-CRP change from baseline as the response and the stratification variables (for region, strata levels were EU vs. NA), visit, treatment, treatment-by-visit interaction and the baseline DAS28-CRP measurement as predictors, and unstructured covariance matrix in the model. DAS28-CRP change from baseline at weeks 8, 12, and 24 are included in the repeated measure analysis

Results of sensitivity analyses of the primary efficacy endpoint using the per-protocol analysis set were consistent with those of the primary efficacy analysis, further confirming the clinical equivalence between ABP 798 and rituximab RP (Table 3). Similar conclusions were drawn from other sensitivity analyses using an ANCOVA adjusting for stratification factors and baseline DAS28-CRP results, analysis exploring the impact of baseline covariates (Table 3), and a tipping point analysis. In addition, subgroup analyses also substantiated the results of the primary analysis for subgroups with larger sample size (i.e., age > 65 years, white race, female, binding ADA positive, binding ADA negative, geographic region of Europe, RF positive and/or CCP positive, 1 prior biologic use, and > 1 prior biologic use).

**Table 3:** Sensitivity analyses of change in DAS28-CRP from baseline at week 24

Sensitivity analysis statistic	ABP 798	Rituximab US + EU	Rituximab US	Rituximab EU
PP analysis set				
*n*	94	191	93	98
Mean (SD)	− 2.207	− 2.123 (1.3287)	− 2.075 (1.3125)	− 2.169 (1.3560)
Difference between means^a^	(1.3726)	0.007	− 0.081	0.093
90% CI		− 0.242, 0.255	− 0.368, 0.207	− 0.193, 0.378
95% CI		− 0.290, 0.303	− 0.424, 0.262	− 0.248, 0.433
ANCOVA (FAS)				
*n*	104	207	103	104
Mean (SD)	− 2.197	− 2.125 (1.3250)	− 2.081 (1.3054)	− 2.168 (1.3491)
Difference between means^b^	(1.3689)	0.035	− 0.061	0.129
90% CI		− 0.209, 0.279	− 0.343, 0.221	− 0.152, 0.409
95% CI		− 0.256, 0.326	− 0.397, 0.275	− 0.206, 0.464
Additional selective covariate analysis (FAS)				
*n*	104	207	103	104
Mean (SD)	− 2.197	− 2.125 (1.3250)	− 2.081 (1.3054)	− 2.168 (1.3491)
Difference between means^c^	(1.3689)	− 0.026	− 0.157	0.107
90% CI		− 0.283, 0.231	− 0.452, 0.139	− 0.189, 0.403
95% CI		− 0.332, 0.280	− 0.509, 0.195	− 0.246, 0.460

*ADA* anti-drug antibodies, *ANCOVA* analysis of covariance, *CI* confidence interval, *DAS28-CRP* Disease Activity Score in 28-joint C-reactive protein, *FAS* full analysis set, *EU* European Union, *NA* North America, *PP* per protocol set, *US* United States

^a^Based on repeated measures analysis with DAS28-CRP change from baseline as the response and the stratification variables region (EU vs. EU), visit, treatment, treatment-by-visit interaction and the baseline DAS28-CRP measurement as predictors, and unstructured covariance matrix in the model

^b^Based on ANCOVA with the DAS28-CRP change from baseline as the response and the stratification variables of region (EU vs. US) and the baseline DAS28-CRP measurement as predictors

^c^Based on a repeated measures analysis with the DAS28-CRP change from baseline as the response and the stratification variables of region (EU vs. NA), visit, treatment, treatment-by-visit interaction, the baseline DAS28-CRP measurement and binding ADA as predictors, and unstructured covariance structure in the model

**Fig. 3:**
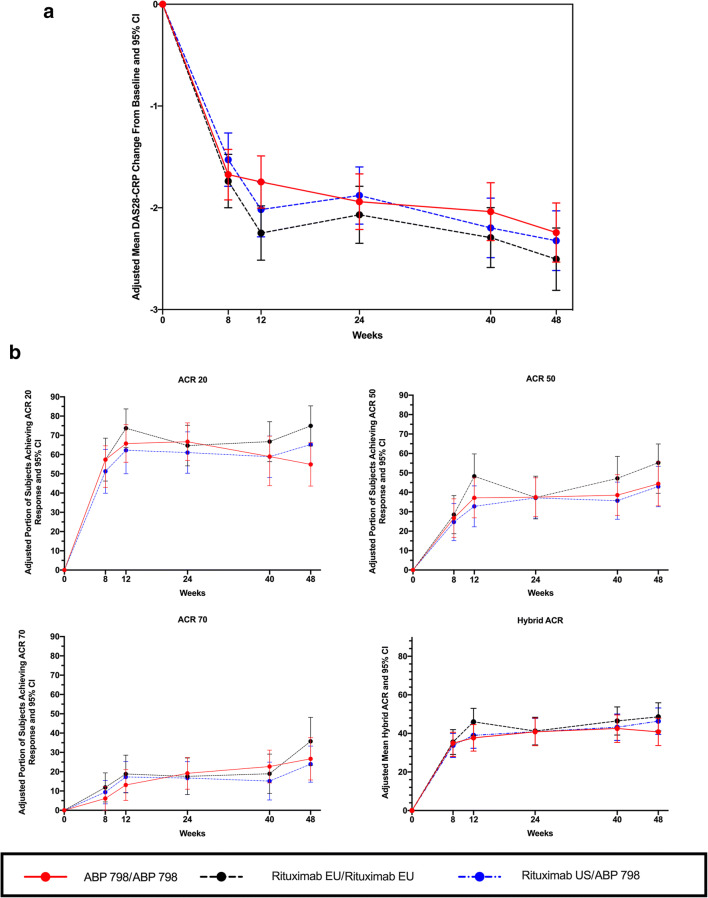
Comparative efficacy of ABP 798 versus rituximab across time (day 1 through EOS). **a** DAS28-CRP change from baseline. **b** Proportion of subjects achieving (a) ACR 20, (b) ACR 50, (c) ACR 70, and (d) hybrid ACR responses (day 1 through EOS). ACR American College of Rheumatology core set measurements, EOS end of study, CRP C-reactive protein, DAS disease activity score, EU European Union, US United States

Mean decreases from baseline in DAS28-CRP were similar across the 3 study groups up to week 48, indicating improvement in disease activity that was maintained through the EOS (Fig. 3a). Over the study period (day 1 to week 48), a similar proportion of subjects achieved ACR20, ACR50, and ACR70 responses in the ABP 798/ABP 798, rituximab EU/rituximab EU, and rituximab US/ABP 798 groups (Fig. 3b). The mean hybrid ACR scores were also comparable across the 3 groups (Fig. 3b). Results from analysis of these secondary efficacy endpoints further supported a conclusion of clinical similarity across treatment groups and also indicated no impact of a single transition on efficacy.

### Safety

All 311 subjects that received IP were included in the safety analysis. The frequency, type, and severity of TEAEs were similar between treatment groups for both the dose periods, i.e., from day 1 to the first infusion of the second dose and from day 1 to EOS (Table 4).

**Table 4:** Overall safety results

	ABP 798/ABP 798 (*N* = 104)	Rituximab EU/Rituximab EU (*N* = 104)	Rituximab US/ABP 798 (*N* = 103)
Day 1 until first infusion of second infusion			
Any adverse event, *n* (%)	52 (50.0)	44 (42.3)	44 (42.7)
Any grade ≥ 3 adverse event, *n* (%)	4 (3.8)	6 (5.8)	4 (3.9)
Any fatal adverse event, *n* (%)	0 (0.0)	0 (0.0)	0 (0.0)
Any serious adverse event, *n* (%)	4 (3.8)	5 (4.8)	5 (4.9)
Any adverse event leading to discontinuation of IP/study, *n* (%)	3 (2.9)	1 (1.0)	4 (3.9)
Adverse events of interest, *n* (%)	19 (18.3)	11 (10.6)	18 (17.5)
Infusion reactions including hypersensitivity	12 (11.5)	7 (6.7)	12 (11.7)
Hematological reactions	4 (3.8)	2 (1.9)	3 (2.9)
Serious infections	2 (1.9)	3 (2.9)	1 (1.0)
Cardiac disorders	2 (1.9)	2 (1.9)	2 (1.9)
Opportunistic infection	1 (1.0)	0 (0.0)	1 (1.0)
Day 1 through end of study			
Any adverse event, *n* (%)	67 (64.4)	54 (51.9)	56 (54.4)
Any grade ≥ 3 adverse event, *n* (%)	5 (4.8)	9 (8.7)	9 (8.7)
Any fatal adverse event, *n* (%)	0 (0.0)	0 (0.0)	0 (0.0)
Any serious adverse event, *n* (%)	8 (7.7)	8 (7.7)	8 (7.8)
Any adverse event leading to discontinuation of IP/study, *n* (%)	3 (2.9)	2 (1.9)	7 (6.8)
Adverse events of interest, *n* (%)	25 (24.0)	15 (14.4)	23 (22.3)
Infusion reactions including hypersensitivity	16 (15.4)	9 (8.7)	16 (15.5)
Hematological reactions	5 (4.8)	2 (1.9)	7 (6.8)
Serious infections	4 (3.8)	4 (3.8)	1 (1.0)
Cardiac disorders	4 (3.8)	3 (2.9)	2 (1.9)
Opportunistic infection	1 (1.0)	2 (1.9)	1 (1.0)

*EU* European Union, *IP* investigational product, *N* number of subjects, *n* number of subjects with event, *US* United States

During the first dose period (day 1 to the first infusion of the second dose), approximately half of the subjects (140 [45.0%]) reported at least 1 TEAE across the 3 groups (ABP 798: 52 [50.0%]; rituximab EU: 44 [42.3%]; rituximab US: 44 [42.7%]) (Table 4). A total of 14 (4.5%) subjects experienced grade ≥ 3 TEAEs (ABP 798: 4 [3.8%]; rituximab EU: 6 [5.8%]; rituximab US: 4 [3.9%]); SAEs were reported in 4 (3.8%), 5 (4.8%), and 5 (4.9%) subjects, respectively. Overall, although there were numerical differences in all grade TEAEs, the majority of TEAEs were grade 1 or 2 in severity and were not thought to be clinically meaningful.

Over the entire study (from day 1 through the EOS), a similar proportion of subjects experienced all grade TEAEs in the 3 groups (ABP 798/ABP 798: 67 [64.4%]; rituximab EU/rituximab EU: 54 [51.9%]; rituximab US/ABP 798: 56 [54.4%]) **(**Table 4). Common AEs reported in ≥ 5% of subjects in any treatment group were upper respiratory tract infection, RA, nasopharyngitis, nausea, and bronchitis.

From day 1 through the EOS, a total of 23 (7.4%) subjects experienced grade ≥ 3 AEs (ABP 798/ABP 798: 5 [4.8%]; rituximab EU/rituximab EU: 9 [8.7%]; rituximab US/ABP 798: 9 [8.7%]) (Table 4). Grade ≥ 3 AEs that occurred in more than 1 subject overall were acute myocardial infarction (1 patient each in the ABP 798/ABP 798 and rituximab EU/rituximab EU groups), back pain (2 patients in the rituximab EU/rituximab EU group), and pneumonia (2 patients in the rituximab EU/rituximab EU group). The incidences of these AEs were similar in the 3 groups.

From day 1 through the EOS, SAEs occurred in 24 subjects and were balanced across the treatment groups (ABP 798/ABP 798: 8 [7.7%]; rituximab EU/rituximab EU: 8 [7.7%]; rituximab US/ABP 798: 8 [7.8]) (Table 4). No deaths were reported in the study. Three (2.9%) subjects in the ABP 798/ABP 798 treatment group, 7 (6.7%) subjects in the rituximab EU/rituximab EU treatment group, and 5 (4.9%) subjects in the rituximab US/ABP 798 treatment group experienced grade ≥ 3 events that were also SAEs. SAEs that occurred in more than 1 subject included acute myocardial infarction, urinary tract infection, back pain, and pneumonia; there were no notable differences in the incidence or severity of SAEs among the 3 groups.

In terms of AEOIs, from day 1 until the EOS, 25 (24.0%) subjects in the ABP 798/ABP 798 group, 15 (14.4%) subjects in the rituximab EU/rituximab EU group, and 23 (22.3%) subjects in the rituximab US/ABP 798 group experienced an AEOI (Table 4). Infusion reactions, including hypersensitivity, were the most common AEOI reported (ABP 798/ABP 798: 16 [15.4%]; rituximab EU/rituximab EU: 9 [8.7%]; rituximab US/ABP 798: 16 [15.5%]). Hypersensitivity AEOIs observed were grade 1 or 2 pruritus, erythema, headache, and rash; no anaphylaxis events were reported. A total of 14 patients experienced hematologic reaction AEOIs (ABP 798/ABP 798: 5 [4.8%]; rituximab EU/rituximab EU: 2 [1.9%]; rituximab US/ABP 798: 7 [6.8%]). The most common of these events was anemia (ABP 798/ABP 798: 3 [2.9%]; rituximab EU/rituximab EU: 0 [0.0%]; rituximab US/ABP 798: 5 [4.9%]). Aside from grade 3 lymphopenia in one patient in the rituximab EU/rituximab EU group, all of the hematologic reaction AEOIs were grade 1 or 2, and none were SAEs. Overall, while numerical differences were observed across treatment groups from day 1 through the EOS, review of the type, nature, and severity of the individual AEOIs did not generate new safety signals compared with those known for the rituximab RP in general**.**

From day 1 through the EOS, AEs led to IP or study discontinuation in 12 subjects (ABP 798/ABP 798: 3 [2.9%]; rituximab EU/rituximab EU: 2 [1.9%]; rituximab US/ABP 798: 7 [6.8%]) (Table 4). Adverse events leading to IP or study discontinuation were mostly infusion reaction events (i.e., hypersensitivity, urticaria, blister, erythema, pruritus, rash, and rash pruritic), which are expected events with rituximab. The numerical differences observed across treatment groups are not considered clinically meaningful, and no safety trends were noted.

Also, following the single transition from rituximab US to ABP 798, the incidences of all grade TEAEs, grade ≥ 3 AEs, SAEs, AEOIs, and AEs leading to IP or study discontinuations were similar across the 3 groups. Any numerical differences between groups in safety, particularly EOIs including the subcategory of infusion reactions, may be attributable to differences that occurred during dose period 1 prior to switching, indicating that the single switch did not impact safety.

### Immunogenicity

Immunogenicity assessments were done for all 311 subjects (Table 5). At baseline, a total of 7 (6.7%) subjects in the ABP 798/ABP 798 group, 10 (9.6%) subjects in the rituximab EU/rituximab EU group, and 6 (5.8%) subjects in the rituximab US/ABP 798 group tested positive for pre-existing binding ADAs; pre-existing neutralizing ADAs were observed in 2 (1.9%), 2 (1.9%), and 3 (2.9%) subjects, respectively.

**Table 5:** Overall immunogenicity results

	ABP 798/ABP 798 (*N* = 97)	Rituximab EU/Rituximab EU (*N* = 94)	Rituximab US/ABP 798 (*N* = 97)
Day 1 until first infusion of second dose^a^			
Developing binding antibody, *n* (%)	13 (13.4%)	10 (10.6%)	19 (19.6%)
Transient	2 (2.1%)	2 (2.1%)	5 (5.2%)
Developing neutralizing antibody, *n* (%)	8 (8.2%)	2 (2.1%)	8 (8.2%)
Transient	0 (0.0%)	0 (0.0%)	0 (0.0%)
Day 1 through end of study^a^			
Developing binding antibody, *n* (%)	14 (14.4%)	13 (13.8%)	20 (20.6%)
Transient	8 (8.2%)	8 (8.5%)	11 (11.3%)
Developing neutralizing antibody, *n* (%)	8 (8.2%)	4 (4.3%)	10 (10.3%)
Transient	7 (7.2%)	2 (2.1%)	5 (5.2%)

*EU* European Union, *N* number of subjects, *n* number of subjects with event, *US* United States

^a^Subjects with a binding negative or no result at baseline and a post-baseline result

Post-baseline, immunogenicity data for the first dose period have previously been reported [12]. From day 1 through the EOS, 14 (14.4%) subjects in the ABP 798/ABP 798 group, 13 (13.8%) subjects in the rituximab EU/rituximab EU group, and 20 (20.6%) subjects in the rituximab US/ABP 798 group developed binding ADAs. Of these, the ADA results were transient (i.e., negative results at the subject’s last time point tested within the study period) for 8 (8.2%), 8 (8.5%), and 11 (11.3%) subjects, respectively. Neutralizing antibodies were detected in 8 (8.2%) subjects in the ABP 798/ABP 798 group, 4 (4.3%) subjects in the rituximab EU/rituximab EU group, and 10 (10.3%) subjects in the rituximab US/ABP 798 groups; the results were transient for 7 (7.2%), 2 (2.1%), and 5 (5.2%) subjects, respectively.

## Discussion

The results presented here demonstrated clinical equivalence of ABP 798 with rituximab RP, based on the 90% CI for the primary efficacy endpoint of DAS28-CRP change from baseline at week 24 being contained within prespecified equivalence margin of − 0.6, 0.6. A change in DAS28-CRP at week 24 was chosen for the primary endpoint to establish clinical equivalence. DAS is a continuous variable that may be sensitive for comparison of 2 similar drug entities such as a proposed biosimilar and the originator RP [19–23]. Additionally, the dichotomous but composite variables of ACR 20/50/70 [22] supported these results. Furthermore, DAS28-CRP also provides correlations with changes in inflammation, disease activity, and radiographic joint damage [24].

In the current study, the conclusion of clinical similarity between ABP 798 and rituximab RP using the continuous variable DAS28-CRP is supported by results from secondary efficacy analyses (DAS28-CRP at other time points; ACR20, ACR50, ACR70, and hybrid ACR). The ACR/EULAR responses and DAS28 scores achieved by both ABP 798 and rituximab RP were comparable with those observed with rituximab RP in originator trials (REFLEX and DANCER trials) [25, 26]. Notably, the primary objective of this comparative clinical study was to demonstrate PK similarity of ABP 798 relative to that of rituximab RP, which has been established and reported separately [12, 13].

The frequency, type, and severity of AEs were similar between treatment groups, with no clinically meaningful differences noted. No new safety signals emerged in this study, with the incidences of AEs falling within the expected range for incidence and severity as previously described for rituximab RP [1, 25, 26]. Infusion reactions including hypersensitivity were the most common AEOIs; however, these were grade 1 or 2 in severity, and none were serious. It must be noted that across all treatment groups, subject incidences of infusion reactions including hypersensitivity AEOIs were highest following the first infusion of the first dose and decreased in subsequent cycles; most resolved during the trial. Overall, although numerical differences were noted across the groups, these are not thought to be clinically meaningful and fell within the expected range of incidence and severity for rituximab RP. In pooled placebo-controlled studies of rituximab RP in RA, infusion-related reactions occurred in 32% of patients within 24 h following their first infusion and occurred in 11% of patients during the 24-h period following the second infusion [1].

In terms of immunogenicity, the development of binding and neutralizing ADAs over the course of the study was comparable across the 3 treatment groups. Importantly, given that clinical responses and safety achieved by both ABP 798 and rituximab RP in this trial are comparable with previously reported clinical trial data for rituximab RP in patients with moderate-to-severe RA, the development of ADAs does not appear to impact clinical efficacy or safety in this patient population.

These results also demonstrate that the single transition from rituximab US to ABP 798 does not result in discernible differences in efficacy, safety, or immunogenicity. From a clinical practice perspective, these data may potentially inform treatment decisions when considering a switch from rituximab RP to ABP 798.

One of the limitations of this study may be the lack of inclusion of DAS28-ESR measurements; however, since this study was designed as a comparative clinical trial for determining similarity of ABP 798 to rituximab RP, this should not impact the overall results. Another limitation may be the lack of a long-term follow-up. However, for biosimilar studies, the key objective is to determine similarity and no clinically meaningful differences between the biosimilar candidate and a reference product. The design, therefore, differs from that of phase 3 clinical trials of novel agents; comparative trials include fewer efficacy assessments and time points. This inclusion of the single switch design enabled confirmation that transitioning from the RP to ABP 798 did not impact immunogenicity. The second 24-week period also enabled further collection of efficacy and safety data. It is important to note that this study design met the criteria for comparative clinical evaluation of biosimilars.

## Conclusions

This double-blind, randomized comparative study confirmed clinical equivalence in terms of efficacy between ABP 798 and rituximab RP in patients with active moderate-to-severe RA. The overall safety and immunogenicity were similar between ABP 798 and rituximab RP over the entire study period and were not affected by the single transition. Together with the report of PK equivalence between ABP 798 and rituximab RP, the current results confirm clinical similarity between the 2 agents. Along with the demonstration of analytical similarity between ABP 798 and the rituximab RP, the results of this comparative clinical study contribute towards the totality of evidence that is required for demonstrating biosimilarity between a proposed biosimilar and the RP.

## Footnotes

1 At the time of this publication, ABP 798 has not been approved by the FDA or other relevant regulatory agency and the indications are yet undetermined. Please consult ABP 798’s later approved label in the relevant country for information regarding the approved uses for ABP 798 in your country.
